# Frequency and functional translation of low muscle mass in overweight and obese patients with COPD

**DOI:** 10.1186/s12931-021-01689-w

**Published:** 2021-03-25

**Authors:** Felipe V. C. Machado, Martijn A. Spruit, Miriam T. J. Groenen, Sarah Houben-Wilke, Paula P. van Melick, Nidia A. Hernandes, Annemie M. W. J. Schols, Fabio Pitta, Emiel F. M. Wouters, Frits M. E. Franssen

**Affiliations:** 1 Department of Research and Development, CIRO+, Centre of Expertise for Chronic Organ Failure, 6080 AB Haelen, Hornerheide 1, Postbus 4009, 6085 NM Horn, The Netherlands; 2 NUTRIM-School of Nutrition and Translational Research in Metabolism, Maastricht, The Netherlands; 3 Department of Respiratory Medicine, Maastricht University Medical Centre (MUMC+), Maastricht, The Netherlands; 4 Laboratory of Research in Respiratory Physiotherapy, Department of Physical Therapy, State University of Londrina, Londrina, Brazil; 5 Ludwig Boltzmann Institute for Lung Health, Vienna, Austria

**Keywords:** Prevalence, Sarcopenia, Obesity, Exercise tolerance, Quality of life

## Abstract

**Background:**

Cut offs for fat-free mass index (FFMI) and appendicular skeletal muscle mass index (ASMI) are available for diagnosing low muscle mass in patients with COPD. This study aimed to investigate: (1) the frequency of low muscle mass (FFMI and ASMI) applying different cut-offs and (2) the functional translation (clinical impact) of low muscle mass, in patients with COPD stratified into BMI categories.

**Methods:**

Patients with COPD were assessed regarding body composition, exercise capacity, quadriceps muscle strength, symptoms of anxiety and depression, dyspnea and quality of life upon referral to pulmonary rehabilitation. The proportion of patients with low muscle mass was compared among BMI categories. Clinical outcomes between patients with normal and low muscle mass within each BMI category were compared.

**Results:**

469 patients with COPD were included for analyses. The frequency of patients classified as low FFMI varied significantly according to the choice of cut-off (32 to 54%; P < 0.05), whereas the frequency of patients with low ASMI was 62%. When applying age-gender-BMI-specific cut-offs, 254 patients (54%) were classified as low FFMI. The choice of the cut-off affected the frequency of patients with low muscle mass in all BMI categories. Overweight and obese patients with low muscle mass were more frequently males and presented worse pulmonary function, exercise capacity and muscle strength compared with overweight and obese patients with normal muscle mass.

**Conclusions:**

Approximately half of the overweight and obese patients with COPD have low muscle mass when applying age-gender-BMI-specific cut-offs. Low muscle mass is associated with worse functional outcomes in overweight and obese COPD patients.

## Introduction

Chronic obstructive pulmonary disease (COPD) is defined by the presence of chronic respiratory symptoms and airflow limitation [1]. Extra-pulmonary features and comorbidities contribute to the burden of this disease [2] and are recognized as treatable traits in the integrated management of the disease [3]. Low fat-free mass (FFM), as a whole-body marker of muscle mass, is commonly found in COPD [4, 5] and strongly associated with muscle weakness [6, 7], exercise intolerance [8] and poor health status [9]. Obesity is another condition frequently coinciding with COPD [10] related to increased respiratory symptoms [11], reduced health status [12] and low functional performance [13].

For the measurement of body composition in COPD, one of the most appropriate methods is dual-energy X-ray absorptiometry (DEXA), which allows a combined assessment of FFM, fat-mass and bone mineral density [14]. In addition, DEXA provides an assessment of FFM and fat-mass at regional level and can provide the measurement of appendicular skeletal muscle mass index (ASMI), which is used to define sarcopenia according to fixed cut-offs < 7.23 kg·m^−2^ for men and < 5.67 kg·m^−2^ for women [15]. However, DEXA is relatively expensive and not widely available. As an alternative, bioelectrical impedance analysis (BIA) is an easy, non-invasive and relatively less expensive method to assess whole-body FFM, widely used in many clinical settings [16, 17]. Despite not enabling assessment of ASMI, BIA can provide an estimate of the whole-body FFM that is usually normalized for body size (dividing FFM for height squared) and expressed as FFM index (FFMI). Irrespective of the methodology of assessment, the European Respiratory Society statement on nutritional management of COPD, proposed a cut-off of 17 kg m^−2^ for males and 15 kg m^−2^ for females to identify patients with low FFMI [14]. These values correspond to the 10th percentile of most normal to underweight patients with COPD [14]. However, it is important to consider that body composition is positively related to body mass index (BMI) and that FFMI declines with aging [18, 19]. Hence, the use of fixed cut-off values may result in underdiagnoses of low FFMI in overweight or obese patients [20, 21] and overdiagnoses in underweight patients and those with advanced age. For underweight (BMI lower than 18.5 kg m^−2^) patients with COPD, the clinical impact of the choice of the cut-off value might be less relevant, since low BMI by itself, provides useful prognostic information [22]; however, this issue is relevant in COPD patients with BMI corresponding to normal weight, overweight and obesity, since BMI is not reliable to determine (ab)normal fat mass and FFM values in these groups [23, 24].

In 2014, Franssen et al. [25] published age-sex-BMI-specific reference values for FFMI based on a sample of 186,975 healthy subjects. The frequency of low FFMI in overweight and obese patients with COPD diagnosed according to age-sex-BMI-specific cut-offs in comparison with fixed cut-off for FFMI and ASMI is currently unknown, as well as whether and to what extent low FFMI is translated in functional impairment in patients in different BMI categories. We hypothesize that the use of age-sex-BMI-specific cut-offs may improve the diagnosis of body composition abnormalities in patients with higher BMI and discriminate groups of patients with more impairment in physical function and clinical characteristics within the category of high BMI patients. Therefore, the aims of the present study were to investigate: (1) the frequency of low muscle mass (FFMI and ASMI) applying different cut-offs and (2) the functional translation (clinical impact) of low muscle mass, in patients with COPD stratified into BMI categories.

## Material and methods

### Study population

The current analysis used data from the Chance Study: an observational, prospective, single-center study focused on COPD, health status and comorbidities [26]. The study was approved by the Medical Ethical Committee of the Maastricht University Medical Centre + (METC 11-3-070) and is registered at http://www.trialregister.nl (NTR 3416). Inclusion criteria were: (1) diagnosis of COPD according to GOLD strategy [1], (2) referral for a comprehensive pulmonary rehabilitation program at CIRO (Horn, the Netherlands) and (3) no exacerbation at least 4 weeks prior to the study. Patients were excluded if they had a history of other lung diseases, had undergone lung surgery or had malignancy within the last five years and/or presented BMI lower than 18.5 kg m^−2^. All patients gave written informed consent, and the study was carried out in accordance with the ethical standards laid down in the 1964 Declaration of Helsinki and its later amendments.

### Procedures

In addition to medical history, anthropometric and demographic variables, DEXA (Lunar Expert-XL Bone Densitometer; Lunar Radiation Corporation, Madison, WI, USA) was performed to assess body composition. FFMI was calculated by dividing FFM (lean mass plus bone mineral content (BMC) by height^2^. ASMI was calculated as the sum of lean mass (minus BMC) for each of the four extremities divided by height^2^. The following measurements were also performed: symptoms of dyspnoea using the modified Medical Research Council (mMRC) dyspnoea scale; exercise capacity using a symptom-limited cardiopulmonary incremental cycle test, the six-minute walking test (6MWT) and a constant work rate exercise test (CWRT); quadriceps peak muscle strength using a isokinetic dynamometer (Biodex System 4 Pro, Biodex Medical Systems, Inc., New York, USA); health related quality of life (HRQL) using St. George’s Respiratory Questionnaire (SGRQ); and symptoms of anxiety and depression using Hospital Anxiety and Depression Scale (HADS).

### Statistical analysis

Patients were classified into BMI categories according to World Health Organization criteria [27]: normal weight (18.5–24.9 kg·m^−2^), overweight (25–29.9 kg·m^−2^) or obese (≥ 30 kg·m^−2^). Afterwards, patients were sub-classified within each BMI category into low or normal FFMI and low or normal ASMI. For FFMI, two cut-offs were applied: the European Respiratory Society statement sex-specific values [14] (17 kg m^−2^ for males and 15 kg m^−2^ for females) and the 10th percentiles of the reference values from the UK Biobank general population (age-sex-BMI-specific cut-offs) [25]. For ASMI classification, the cut-offs applied (< 7.23 kg·m^−2^ for men; < 5.67 kg·m^−2^ for women) were in accordance with the Health Aging and Body Composition (Health ABC) Study [15].

Continuous variables are presented as mean and standard deviation (SD) or median [interquartile range 25–75%], according to normality in data distribution. Categorical variables are presented as absolute and relative frequency. A chi-square test of independence was conducted to investigate whether there is association between the choice of different cut-offs and the proportion of patients diagnosed with low FFMI and ASMI. The comparisons of continuous variables between patients with normal or low FFMI and normal or low ASMI within each BMI category were performed with Student’s t-test for independent samples or Mann–Whitney U-test, whereas the comparisons of categorical variables were performed with a Chi-square test. Statistics were performed using SPSS (version 24.0, IBM Corporation, Armonk, NY, USA). A priori, the level of significance was set at P < 0.05.

## Results

The Chance study enrolled 518 patients with COPD. Nineteen patients were excluded from the analysis because of missing body composition analysis and 30 patients were excluded due to BMI < 18.5 kg·m^−2^. The general characteristics of the included patients are presented in Table 1. Patients were on average 64 years and presented severe airflow limitation, reduced exercise capacity and quadriceps muscle strength and impaired HRQL. More than half of patients were overweight or obese.

**Table 1:** Characteristics of COPD patients with normal and low FFMI according to age-sex BMI-specific cut-offs, after stratification into BMI categories

BMI group	Normal weight (n=206)		Overweight (n=157)		Obese (n=106)	
FFMI group	Normal (n=88)	Low (n=118)	Normal (n=66)	Low (n=91)	Normal (n=61)	Low (n=45)
Subjects, % males	49.0	52.5	50.0	67.0*	44.3	66.7*
Age, y	64 ± 10	64 ± 8	65 ± 9	65 ± 10	64 ± 8	64 ± 9
BMI, kg m^-2^	22.3 ± 1.8	21.6 ± 1.7†	27.7 ± 1.5	27.0 ± 1.2†	34.4 ± 2.9	31.7 ± 1.8#
FFMI, kg m^-2^	17.2 [15.2–18.0]	14.5 [13.8–16.3]#	19.0 [16.4–19.6]	17.1 [15.5–18.2]#	20.4 [18.6–21.8]	19.1 [16.5–20.1]#
FEV_1_, l	1.10 [0.83–1.44]	0.92 [0.68–1.41]	1.33 [0.96–1.90]	1.13 [0.77–1.72]	1.32 [0.99–1.74]	1.45 [1.04–1.92]
FEV_1_, %pred	44.5 [34–55.6]	37.3 [27.4–51.1]†	55.6 [41.9–69.4]	41.0 [32.0–65.7]*	56.0 [41.4–70.3]	53.5 [42.6–63.2]
FEV_1_/FVC, %	32.6 [27.8–41.3]	30.8 [25.2–41.2]	40.3 [32.0–48.2]	33.0 [25.6–44.7]*	44 [34.7–53.4]	41.6 [33.5–49.2]
TLCO, %pred	45.5 [37–57.8]	40.3 [32.0–48.5]†	46.6 [38.0–63.0]	48.1 [40.9–61.1]	57.3 [49.5–68.8]	50.8 [42.0–60.1]*
mMRC, % grade ≥ 2	70.4	87.3†	81.8	75.8	86.7	82.2
6MWD, m	458 ± 121	410 ± 122†	434 ± 118	414 ± 126	402 ± 108	430 ± 118
6MWD, %pred	70 ±17	62 ± 18#	70 ± 17	67 ±17	70 ± 17	69 ± 16
Wmax, W	63 [49–87]	53 [41–68]†	69 [54–97]	64 [48–83]	66 [55–86]	75 [54–97]
Wmax, %pred	52 [38–73]	42 [31–61]†	55 [44–76]	48 [37–64]*	59 [41–91]	53 [43–65]
VO_2_max, ml m^-1^	998 [805–1234]	811 [661–978]#	1108 [872– 1349]	1088 [900–1291]	1126 [892–1368]	1267 [974–1542]
VO_2_max, %pred	57 [47–78]	50 [36–73]*	67 [51–86]	58 [48–77]	81 [55–112]	64 [54–75]*
CWRT, s	267 [184–351]	190 [149–269]#	250 [173–343]	218 [176–302]	243 [172–445]	254 [171–410]
Quadriceps PT, Nm	87 ± 26	77 ± 29†	105 ± 37	95 ± 32	107 ± 38	108 ± 42
Quadriceps PT, %pred	64 ± 17	55 ± 14#	75 ± 17	65 ± 18†	79 ± 15	72 ± 21*
SGRQ Total score, pts	59 [39–75]	63 [50–75]	62 [49–73]	63 [49–73]	68 [57–75]	63 [52–73]
HADS Anxiety score, pts	7 [4–11]	6 [4–11]	8 [5–13]	6 [4–10]	7 [5–12]	8 [4–12]
HADS Depression score, pts	7 [3–11]	6 [3–10]	7 [4–11]	7 [4–10]	8 [5–10]	6 [4–11]
ASMI, kg m^-2^	6.58 ± 0.80	5.73 ± 0.77*	7.18 ± 1.06	6.67 ± 0.91*	7.98 ± 1.13	7.50 ± 1.10*
Lumbar spine, T-score	− 1.29 ± 1.38	− 1.09 ± 1.69	− 0.74 ± 1.37	− 0.99 ± 1.66	− 0.68 ± 1.27	− 0.87 ± 1.64
Hip, T-score	− 1.56 ± 1.00	− 1.86 ± 0.95*	− 1.69 ±0.90	− 1.67 ± 1.00	− 1.15 ± 1.06	− 1.19 ± 0.91

Mean ± standard deviation, median [interquartile range 25–75%] or frequency reported. *ASMI* appendicular skeletal muscle mass index; *BMI* body mass index; *FFMI* fat-free mass index; *FEV*_*1*_ forced expiratory volume in the first second; *FVC* forced vital capacity; *TLCO* transfer factor for carbon monoxide; *mMRC* modified Medical Research Council; *6MWD* six-minute walking distance; *Wmax* peak load during cycle ergometry; *VO*_*2*_*max* peak oxygen uptake during cycle ergometry; *CWRT* time during constant work rate test; *PT* peak torque; *SGRQ* St George’s Respiratory Questionnaire; HADS hospital anxiety and depression questionnaire. *P < 0.05 versus normal FFMI from the same BMI group. †*P* < 0.01 versus normal FFMI from the same BMI group. #*P* < 0.001 versus normal FFMI from the same BMI group

### Frequency of low FFMI

**Fig. 1:**
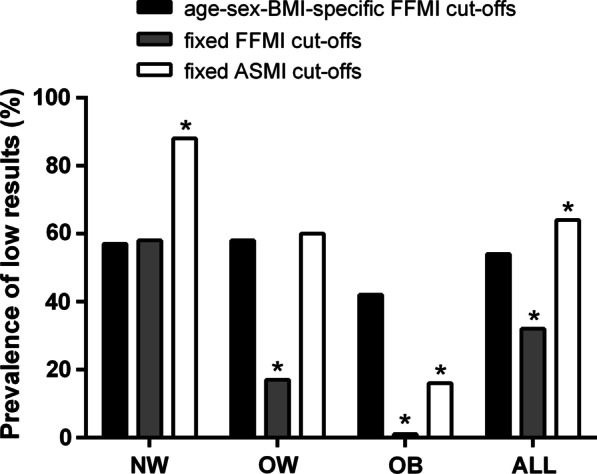
Proportion of patients with low muscle mass, using different cut-offs, after stratification for body mass index. *NW* normal weight, *OW* overweight, *OB* obese, *ALL* all patients, *Chi-square test P < 0.05 vs age-sex-BMI-specific cut-offs

Figure 1 shows the frequency of low FFMI according to the different cut-offs. The overall frequency of patients classified as low FFMI was lower when applying the fixed cut-off in comparison with the age-sex-BMI-specific cut-off (32% and 54%, respectively; *P* < 0.05).

Considering the fixed cut-off, the frequency of low FFMI decreased with an increase in BMI; the frequency of patients with low FFMI in normal weight, overweight and obese categories was 58%, 17% and 1%, respectively. Considering the age-sex-BMI-specific cut-offs, the frequency of patients with low FFMI, in normal weight, overweight and obese groups was 57%, 58% and 42%, respectively. The frequency of low FFMI as identified by the fixed cut-off was comparable to the frequency identified by the age-sex-BMI-specific cut-off for patients with a normal weight BMI, but lower for patients with an overweight or obese BMI (*P* < 0.05, for all) (Fig. 1).

### Clinical impact of low FFMI

Table 1 presents the comparisons of outcomes between patients with normal and low FFMI, according to the age-sex-BMI specific cut-offs, after stratification into BMI categories. A higher frequency of males with low FFMI were found in patients with an overweight or obese BMI. In patients with normal weight, those with low FFMI presented lower forced expiratory volume in the first second (FEV_1_), transfer factor for carbon monoxide (TL_CO_), six-minute walking distance (6MWD), peak load during cycle ergometry (Wmax), peak oxygen consumption during cycle ergometry (VO_2max_), quadriceps peak torque (PT) and hip bone mineral density (BMD) compared with normal weight patients with normal FFMI. Overweight patients with low FFMI presented lower FEV_1_, Wmax and PT, compared with overweight patients with normal FFMI. Finally, obese patients with low FFMI presented lower TL_CO_, VO_2max_ and PT compared to patients with normal FFMI from the same BMI category.

### Frequency of low ASMI

The overall frequency of patients classified as low ASMI was 62% (Fig. 1). The frequency of patients with low ASMI in normal weight, overweight and obese groups was 88%, 60% and 16%, respectively. The frequency of low ASMI was significantly higher than the frequency of low FFMI (according to the age-sex-BMI adjusted cut-offs) in normal weight patients, comparable in overweight patients, and lower for patients stratified into the obese category *(P* < 0.05, for all) (Fig. 1).

### Clinical impact of low ASMI

Comparisons of outcomes between patients with normal and low ASMI after stratification into BMI categories are presented in Table 2. In patients with normal weight, those with low ASMI presented lower FEV_1_, TL_CO_, 6MWD, Wmax, VO_2max_, CWRT, quadriceps PT, lumbar BMD, and a higher proportion of this group were males and patients with symptoms of dyspnea (mMRC ≥ 2) *(P* < 0.05, for all). Considering the overweight patients, those with low ASMI presented lower FEV_1_, 6MWD, Wmax, VO_2max_, quadriceps PT. For the group of obese patients, those with low ASMI presented lower FEV_1_, TL_CO_, 6MWD, Wmax, VO_2max_, quadriceps PT, and higher symptoms of dyspnea.

**Table 2:** Characteristics of COPD patients referred for pulmonary rehabilitation with normal and low ASMI, after stratification into BMI categories

BMI group	Normal weight (n=205)		Overweight (n=156)		Obese (n=103)	
ASMI group	Normal (n=25)	Low (n=180)	Normal (n=62)	Low (n=94)	Normal (n=87)	Low (n=16)
Subjects, % males	28.0	54.4*	53.2	63.8	55.1	56.2
Age, years	62 ± 9	64 ± 9	65 ± 10	66 ± 9	64 ± 9	63 ± 7
BMI, kg m^-2^	21.9 ± 1.9	21.9 ± 1.8	27.8 ± 1.5	27.0 ± 1.2#	33.5 ± 2.9	31.7 ± 2.1#
FFMI, kg m^-2^	16.3 [15.3–18.0]	15.3 [14.2–17.0]†	18.5 [16.5–19.6]	17.1 [15.7–18.3]#	20.1 [18.4–21.4]	18.0 [15.6–19.2]#
FEV_1_, l	1.34 [0.91–1.84]	0.98 [0.74–1.37]†	1.47 [0.96–2.01]	1.09 [0.77–1.57]†	1.49 [1.09–1.83	1.01 [0.66–1.60]†
FEV_1_, %pred	53.2 [43.4–67.1]	37.8 [29.1–52.1]#	59.8 [41.9–74.4]	42.4 [30.9–59.4]#	57.0 [44.6–68.8]	41.3 [27.3–54.5]†
FEV_1_/FVC, %	38.3 [32.1–43.3]	31.0 [25.4–40.8]#	43.4 [32.5–50.7]	32.6 [25.5–43.0]†	44.0 [36.6–52.7]	33.5 [28.1–41.3]†
TLCO, %pred	52.0 [41.5–69.7]	42.1 [33.4–50.0]†	52.0 [40.8–63.1]	47.0 [36.7–61.0]	57.1 [49.2–68.5]	42.0 [34.3–49.0]#
mMRC, % grade ≥ 2	56.0	83.8†	75.8	82.4	82.6	93.7
6MWD, m	523 ± 92	417 ± 123#	471 ± 110	391 ± 122#	426 ± 114	356 ± 97*
6MWD, %pred	81 ± 12	63 ± 18#	76 ± 15	64 ± 17#	71 ± 16	59 ± 15†
Wmax, W	84 [61–98]	55 [44–74]#	74 [58–100]	64 [46–78]†	74 [61–95]	54 [37–72]*
Wmax, %pred	69 [46–96]	45 [32–65]#	58 [48–84]	47 [37–64]#	58 [43–86]	45 [36–55]*
VO_2_max, ml m^-1^	1244 [880–1438]	888 [691–1034]#	1156 [900–1402]	1060 [873–1303]	1230 [995–1501]	958 [818–1147]*
VO_2_max, %pred	75 [51–102]	52 [37–72]#	67 [53–88]	56 [46–76]†	67 [55–97]	55 [46–70]
CWRT, s	285 [219–491]	206 [154–298]†	255 [187–360]	214 [175–314]	258 [172–461]	230 [172–304]
Quadriceps PT, Nm	90 ± 23	81 ± 29	139 ± 35	144 ± 29	113 ± 42	93 ± 29
Quadriceps PT, %pred	72 ± 18	58 ± 15#	76 ± 16	65 ± 17#	79 ± 18	64 ± 13*
SGRQ Total score, pts	55 [38–68]	63 [49–75]	58 [46–70]	65 [52–74]	65 [52–75]	66 [61–79]
HADS Anxiety score, pts	9 [3–11]	6 [4–11]	6 [5–12]	7 [5–11]	7 [5–11]	11 [5–13]*
HADS Depression score, pts	7 [4–11]	6 [3–10]	7 [4–10]	8 [4–11]	8 [4–10]	6 [4–14]
ASMI, kg m^-2^	6.39 ± 0.80	5.55 ± 0.79#	6.93 ± 0.86	6.07 ± 0.87#	7.53 ± 1.00	6.20 ± 0.76
Lumbar spine T-score	− 2.04 ± 1.60	− 1.05 ± 1.53†	− 0.88 ± 1.35	− 0.90 ± 1.67	− 0.74 ± 1.46	− 0.98 ± 1.41
Hip T-score	− 1.66 ± 0.99	− 1.75 ± 0.98	− 1.66 ± 0.89	− 1.70 ± 0.98	− 1.11 ± 1.03	− 1.41 ± 0.80

Mean ± standard deviation, median [interquartile range 25–75%] or frequency reported. *ASMI* appendicular skeletal muscle mass index, *BMI* body mass index, FFMI fat-free mass index, *FEV*_*1*_ forced expiratory volume in the first second, *FVC* forced vital capacity, *TLCO* transfer factor for carbon monoxide, *mMRC* modified Medical Research Council, *6MWD* six-minute walking distance, *Wmax* peak load during cycle ergometry, *VO*_*2*_*max* peak oxygen uptake during cycle ergometry, *CWRT* time during constant work rate test, *PT* peak torque, *SGRQ* St George’s Respiratory Questionnaire, *HADS* hospital anxiety and depression questionnaire, *P < 0.05 versus normal FFMI from the same BMI group. †*P* < 0.01 versus normal FFMI from the same BMI group. #*P* < 0.001 versus normal FFMI from the same BMI group

## Discussion

This study compared the frequency of abnormal body composition diagnosed according to fixed whole-body (FFMI) and regional (ASMI) cut-offs versus age-sex-BMI-specific cut-offs for FFMI in patients with COPD, after stratification into BMI categories. The study has three main findings. First, low FFMI is more commonly diagnosed in overweight and obese patients with COPD using age-sex-BMI-specific cut-offs, in contrast to when fixed cut-offs are applied. Second, the effects of low FFMI are less pronounced in higher categories of BMI, but patients with low FFMI in overweight/obese categories are characterized by worse lung function, muscle strength and exercise tolerance compared to patients with comparable BMI and normal FFMI. Finally, the frequency of males with low FFMI in overweight/obese was higher, despite the use of a sex-specific cut-off, suggesting that sex-dependent FFMI disturbances in these groups of patients.

The first study to apply age-sex-BMI-specific cut-offs for FFMI [23] found that patients with COPD were 3 times more likely to present sarcopenic obesity compared with a control group and that the presence of sarcopenic obesity was associated with worse physical performance and higher systemic inflammation. Despite identifying participants with relative imbalance in fat and FFM across a wide range of BMI [23], this study did not compare the frequency of low FFMI and ASMI according to different cut-offs or the impact of presenting low FFMI after stratification into BMI categories. Another study found that the frequency of patients with low FFMI according to a fixed cut-off was 34.5%. However, from the total sample with low FFMI, 36%, 53% and 11% were underweight, normal weight and overweight, respectively, whereas no obese patient presented low FFMI [21]. Similarly, a previous study which aimed to identified distinct clusters based on the comorbidity profiles in a cohort of moderate to very severe patients with COPD, found that the frequency of low FFMI was 28%, but the metabolic cluster, characterized by a higher frequency of obesity (61%), presented no patients classified as low FFMI (according to fixed cut-offs values) [20]. The study of van de Bool et al. [21] applied the fixed cut-offs for ASMI and found a high frequency of low ASMI across all BMI categories (100%, 97%, 88% and 54% in underweight, normal weight, overweight and obese, respectively). The explanation for the higher frequency of low ASMI in overweight and obese patients in that study compared to the current is unclear as age, sex distribution, disease severity, study center and methodology to assess body composition were comparable.

In addition to further identification of patients with low FFMI, this study also demonstrates the functional translation of low FFMI in patients with higher BMI. We found that the differences in outcomes between overweight/obese patients with normal and low FFMI were less pronounced when compared with the differences observed in normal weight patients, suggesting a lower influence of presenting low FFMI in patients with higher BMI. Our hypothesis is that the direct effects of increased BMI on respiratory mechanics at rest and during exercise could be related with relatively preserved lung function and functional outcomes [28]. In addition, despite FFMI being strongly related with muscle strength, other determinants of strength (e.g., muscle activation, specific force of the muscle fibers) [29] could be enhanced in lower limbs of patients with higher BMI, due to training effect for being constantly submitted to overload during activities of daily living (e.g. walking, climbing stairs). This is supported by findings from the study of van de Bool et al. [21] whose results show that muscle strength increases linearly with an increasing BMI and that patients with low FFMI and abdominally obese (i.e. higher BMI) present higher efficiency of the lower limbs muscles (expressed as the ratio between muscle strength and ASMI).

Exercise and nutrition-based interventions as part of comprehensive pulmonary rehabilitation program should focus not only on treating the deleterious effects of obesity, but also on maintaining or increasing FFM, lower-limb muscle function and exercise tolerance in these patients. In obese patients with COPD, a previous study showed that caloric restriction with maintained protein intake associated with resistance exercise training is effective to promote weight loss, without the loss of muscle mass and with improvement in functional outcomes [30]. These benefits have also been demonstrated in obese older adults, however with additional effects of including aerobic training to calorie restriction and resistance training [31].

In the present study there were no differences in HRQL between patients with normal and low FFMI according to the age-sex-BMI-specific cut-offs. This finding is in contrast with previous studies which showed that patients with low FFMI present worse HRQL, as assessed by using the SGRQ [32, 33]. However, it is not yet clear if the fact of presenting low FFMI is independently associated with reduction of quality of life, since in both studies, other variables, such as dyspnea [32] and exercise capacity [33] were deemed to be mediators of the effect of low FFMI on HRQL. In the present study quality of life was, in general, impaired in patients with COPD, independently of body weight and FFMI categories. Pulmonary rehabilitation is strongly recommended to improve HRQL in patients with COPD and evidence support that patients with low FFMI can improve HRQL to the same extent as patients with normal FFMI [33].

The present study included patients with COPD referred for pulmonary rehabilitation. Therefore, the observed frequency of low FFMI is probably higher compared to the general COPD population. However, rather than establishing the exact frequency of low FFMI in patients with COPD or compare the agreement of different cut-offs, the focus of this study was on the comparison of applying age-sex-BMI-specific and fixed cut-offs to the same cohort of patients for diagnosing low FFMI and ASMI and to provide a better understanding on the effects of low FFMI in different BMI categories. Moreover, in the present study DEXA was used to calculate FFMI, while normative values for FFMI were based on BIA [25]. BIA may lead to a slight underestimation of FFM when compared with DEXA in patients with COPD [34]. Although the 10^th^ percentile values for FFMI based on BIA may represent an even lower percentile of FFMI based on DEXA, this would result in a slightly underestimation of the proportion of patients with low FFMI and the frequency of low FFMI would be actually higher than presented. In addition, the ERS statement on nutritional management in COPD does not mention or recommend the use of method-specific reference values [14]. Thus, the use of age-sex-BMI-specific reference values has shown potential to improve the diagnosis of body composition abnormalities in patients with higher BMI, mainly in clinical practice, considering that BIA is more commonly available than DEXA.

While this study showed that large proportion of overweight and obese COPD patients suffer from low FFMI and its functional consequences, it is not fully understood whether and to what extent these patients benefit from non-pharmacological treatment. Studying the effects of exercise training in combination with nutritional support in overweight and obese patients with low FFMI is an interesting topic for future investigation. Furthermore, the prognostic value and impact of low FFMI on long-term outcomes in overweight and obese patients should be investigated. Finally, longitudinal changes in body composition in these sub-group of patients with COPD and their impact on outcomes can also be part of future research projects.

## Conclusion

This study showed that the application of age-sex-BMI-specific cut-offs resulted in a high proportion of overweight and obese patients with COPD presenting low FFMI and these patients are characterized by worse lung function, muscle strength and exercise tolerance. While it was previously reported that low FFMI is absent in overweight and obese patients with COPD, the present study encourages the application of age-sex-BMI-specific cut-offs in order to identify these patients. The results of the present study have important consequences for the assessment of overweight and obese patients with COPD.

## Data Availability

The datasets used and/or analysed during the current study are available from the corresponding author on reasonable request.

## Abbreviations

COPD: Chronic obstructive pulmonary disease
FFMI: Fat-free mass index
ASMI: Appendicular skeletal muscle mass index
BMI: Body mass index
FFM: Fat-free mass
DEXA: Dual-energy X-ray absorptiometry
BIA: Bioelectrical impedance analysis
BMC: Bone mineral content
mMRC: Modified Medical Research Council
6MWT: Six-minute walking test
CWRT: Constant work rate exercise test
HRQL: Health related quality of life
SGRQ: St. George’s Respiratory Questionnaire
HADS: Hospital Anxiety and Depression Scale
FEV_1_: Forced expiratory volume in the first second
TL_CO_: Transfer factor for carbon monoxide
6MWD: Six-minute walking distance
Wmax: Peak load during cycle ergometry
VO_2max_: Peak oxygen consumption during cycle ergometry
PT: Peak torque
BMD: Bone mineral density
